# Comparative Genomics of Color Morphs In the Coral *Montastraea cavernosa*

**DOI:** 10.1038/s41598-017-16371-9

**Published:** 2017-11-22

**Authors:** Jessica K. Jarett, Matthew D. MacManes, Kathleen M. Morrow, M. Sabrina Pankey, Michael P. Lesser

**Affiliations:** 1 Molecular, Cellular and Biomedical Sciences, University of New Hampshire, Durham, NH 03824 USA; 2 School of Marine Science and Ocean Engineering, University of New Hampshire, Durham, NH 03824 USA; 3 Present Address: US Department of Energy, Joint Genome Institute, 2800 Mitchell Drive, Walnut Creek, CA 94598 USA

## Abstract

*Montastraea cavernosa* is a common coral in the Caribbean basin found in several color morphs. To investigate the causes for brown and orange morphs we undertook a genomics approach on corals collected at the same time and depth in the Bahamas. The coral holobiont includes the host, symbiotic dinoflagellates (*Symbiodinium* spp.), and a diverse microbiome. While the coral host showed significant genetic differentiation between color morphs both the composition of the *Symbiodinium* spp. communities and the prokaryotic communities did not. Both targeted and global gene expression differences in the transcriptome of the host show no difference in fluorescent proteins while the metatranscriptome of the microbiome shows that pigments such as phycoerythrin and orange carotenoid protein of cyanobacterial origin are significantly greater in orange morphs, which is also consistent with the significantly greater number of cyanobacteria quantified by 16S rRNA reads and flow cytometry. The microbiome of orange color morphs expressed significantly more nitrogenase (*nif*H) transcripts consistent with their known ability to fix nitrogen. Both coral and *Symbiodinium* spp. transcriptomes from orange morphs had significantly increased expression of genes related to immune response and apoptosis, which may potentially be involved in maintaining and regulating the unique symbiont population in orange morphs.

## Introduction

The worldwide decline of coral reefs has been attributed to a variety of natural and anthropogenic stressors^1–3^ whose effects appear to be long-term, and potentially irreversible^3–5^. These issues are particularly serious in the Caribbean Basin, where 70% of the coral reefs are already listed as threatened, critically-endangered, or have undergone a phase shift to algal-dominated coral refs^6,7^. The multiple stressors associated with climate change^8^ may represent a “tipping point” for coral reefs that are already severely impacted by hurricanes, overfishing, eutrophication, coastal development and invasions by non-native species^1,9,10^. The need to further understand basic coral biology has never been more important as scleractinian corals are perhaps one of the most diverse symbiotic “ecosystems” in the marine realm^11^. This will require an in depth taxonomic and functional understanding of all of the partners in the holobiont^12^ as well as any interactions. It is complicated by the fact that the number of partners involved includes the cnidarian host, their dinoflagellate endosymbionts (*Symbiodinium* spp.), Bacteria, Archaea, and viruses of all these components, an endolithic community and several recently described Alveolata that all likely have multiple, and potentially interactive, roles in the biology of corals^11–14^.

In the Caribbean, the great star coral *Montastraea cavernosa* Linnaeus is abundant on Caribbean and Eastern Atlantic coral reefs and has an extremely wide depth range from shallow through mesophotic depths (3–100 m)^15^. It is commonly found in three color morphs, cyan, orange and brown^16,17^ and based on multiple lines of molecular and biophysical evidence, the orange morphs contain significant populations of symbiotic cyanobacteria expressing phycoerythrin in their microbiome^18–20^. It is also known that the orange morph of *M*. *cavernosa* expresses a photo-convertible fluorescent protein that contributes to the orange color^16,17^ and it can be difficult to separate these sources of fluorescence based on spectral characteristics that are very similar between the two chromoproteins. Adaptive coloration patterns are most often associated with a warning coloration in predator-prey interactions, also known as aposematic coloration. In marine environments, aposematic coloration appears to be common where visual predators (e.g., fish) are predating upon chemically defended prey^21^. In a previous study, we asked if orange morphs were more effective at deterring visual predators, and if they contained chemical defenses to deter corallivory^22^. Both color morphs are chemically defended from fish predators, but it was shown that aposematic coloration is not occurring in this species. We also know that nitrogen-fixing bacteria, both cyanobacteria and heterotrophs, are present in the host tissues of *Montastraea cavernosa* ^20^ as well as many other corals^23^, and might contribute to other ecologically relevant differences between color morphs. In particular for orange fluorescing *M*. *cavernosa* the bulk stable isotopic nitrogen signatures clearly show that fixed nitrogen is transferred to the symbiotic *Symbiodinium* spp. These symbionts also exhibit a higher growth rate, but maintain a constant density, when compared to colonies without symbiotic cyanobacteria and therefore maintain a stable symbiotic relationship^19^. Coral reefs are classically thought of as nitrogen-limited ecosystems, so it has been hypothesized that the input of “new” nitrogen from nitrogen fixation could have significant positive benefits for the holobiont on oligotrophic reef environments^24–26^. For *M*. *cavernosa* the orange and brown color morphs occur side by side in the same environment, which creates a natural comparative experiment to investigate multiple aspects of the biology of host and microbiome in this species of coral.

In this study, we assess the community composition of the microbiome for both color morphs of *Montastraea cavernosa* using gene-targeted amplicon sequencing, and potential physiological differences in the host and microbiome using RNASeq based transcriptomics. We ask two specific questions: (1) do the molecular and microbial profiles of the two primary color morphs of *M*. *cavernosa* differ as it relates to the expression of genes potentially responsible for their color, and (2) are there differences in global gene expression patterns for the coral host, symbiotic dinoflagellates (i.e., *Symbiodinium* spp.) and microbiome of the holobiont between the color morphs?

## Results

Pyrosequencing of the 16S rRNA gene recovered a total of 186,663 sequences after quality filtering and removal of singletons with an average (±SE) of 13,368 ± 2,098 from orange morphs, 18,461 ± 1,969 from brown morphs and 30,394 ± 2,965 from seawater samples (DRYAD doi:10.5061/dryad.v2g01). A total of 567 unique operational taxonomic units (OTUs) were identified after clustering at 97% and removal of chimera and chloroplast sequences (124 OTUs; 1259 and 1156 total reads in brown and orange morphs respectively). The number of unique OTUs ranged from 264–898 in coral samples and 388–520 in seawater samples. All water samples were sequenced to saturation. Rarefaction curves of observed OTUs approached an asymptote for all coral samples, which were rarefied at a common depth of 2,200 reads for statistical analysis.

**Figure 1:**
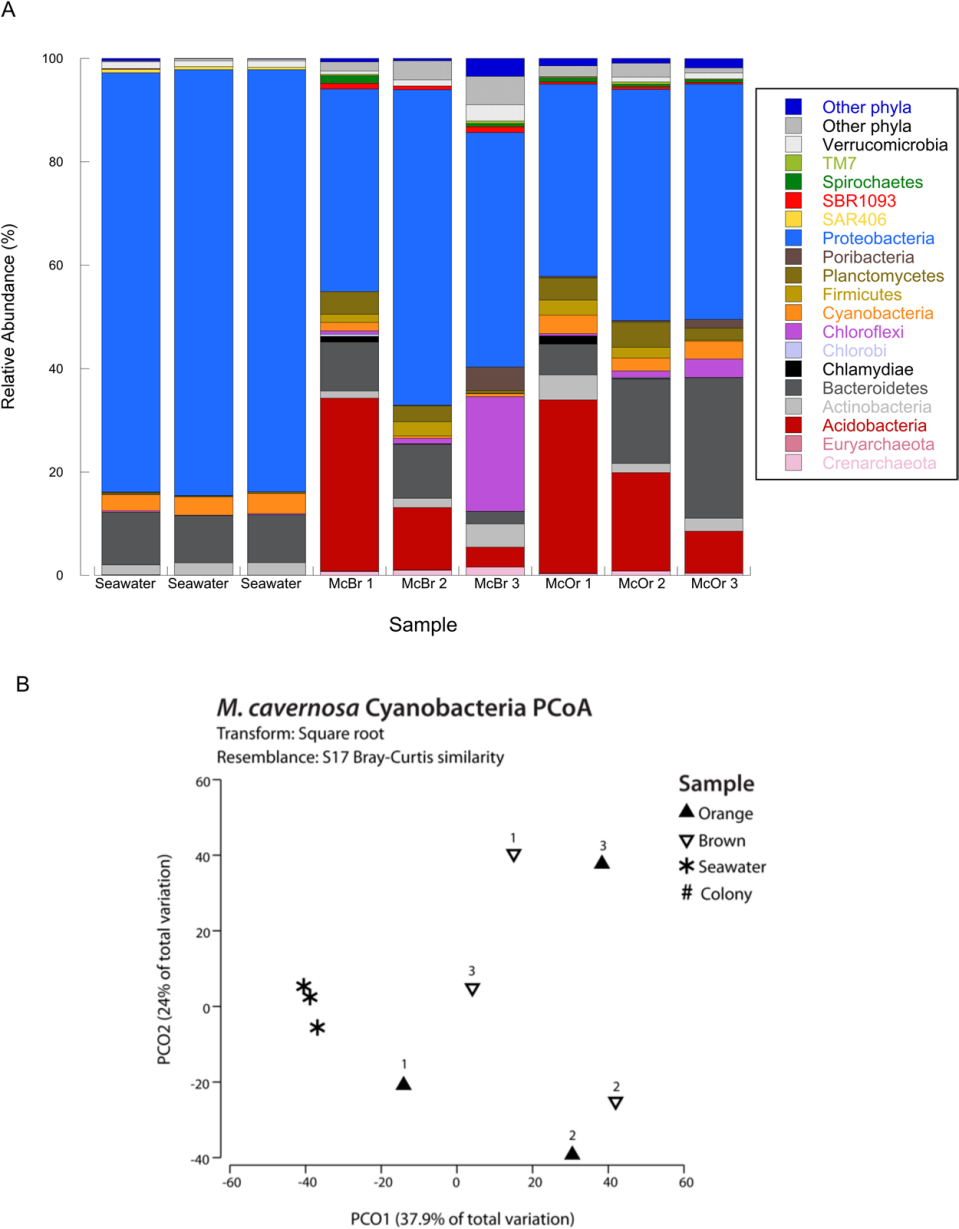
(**A**) Bar graph representing average percent contribution of the most dominant prokaryotic phyla relative to the total abundance in the color morphs of *Montastraea cavernosa* compared to the surrounding ambient seawater. McBr = brown morphs and McOr = orange morphs. (**B**) Principal coordinate analysis (PCoA) plot based on square-root transformed Bray-Curtis similarity for seawater and color morphs of *Montastraea cavernosa*.

Seawater samples were dominated by the phyla *Proteobacteria*, *Cyanobacteria*, *Bacteroidetes*, and *Actinobacteria*, while communities in coral samples were more diverse than seawater samples and exhibited more variability between replicate samples of the same color morph, especially the brown morph (Fig. 1A). In general, prokaryotic communities in the coral samples were dominated by the phyla *Proteobacteria*, *Bacteroidetes*, *Acidobacteria*, *Planctomycetes*, *Cyanobacteria* and *Verrucomicrobia*, with up to 26 other phyla present in low abundance. Coral samples also contained several phyla that were rare or absent in seawater samples, including *Euryarchaeota, Crenarchaeaota*, *Acidobacteria*, *Chlamydiae*, *Chlorobi*, *Chloroflexi*, *Firmicutes*, *Planctomycetes*, and Candidatus *Poribacteria*. *Proteobacteria* dominated both seawater and coral samples, but the composition of proteobacterial orders was very different. Seawater samples were primarily composed of α-*Proteobacteria* including *Rickettsiales* and *Pelagibacteriaceae*, as well as *Halomonadacaea* and other γ-*Proteobacteria*. *Endozoicimonaceae* were found exclusively in coral samples, and *Rhizobiales*, *Pseudoalteromonadaceae*, and *Vibrionaceae* were more abundant in coral samples than seawater samples.

There were significant differences in community structure between corals and the ambient seawater (PERMANOVA, Pseudo-F = 3.62, *P* (perm) = 0.024). Pairwise testing showed that the microbial communities in brown and orange morphs of *Montastraea cavernosa* were not significantly different from each other (Orange vs Brown, PERMANOVA, *t* = 0.932, *P* (perm) = 0.504), but were significantly different than seawater samples (Seawater vs Orange, PERMANOVA, *t* = 2.57, *P* (perm) = 0.011, Seawater vs Brown, PERMANOVA, *t* = 2.41, *P* (perm) = 0.016). *Cyanobacteria* were well-represented taxonomically in coral samples with 62 OTUs but it represented only 0.57 to 3.97% of total reads recovered and there was no significant difference in the community structure of *Cyanobacteria* between orange and brown morphs of *M*. *cavernosa* (PERMANOVA, *t* = 0.99, *P* (perm) = 0.497) after a Bonferroni correction to avoid a Type 1 error using a *P* ≤ 0.025 significance cutoff. A PCoA analysis showed no pattern of community differences for cyanobacterial OTUs except for the distinctiveness of seawater versus all coral samples (Fig. 1B). No cyanobacterial OTUs were found consistently, or exclusively, in orange colonies. Despite this, there was a significant effect of sample type (corals vs. seawater) on the log-transformed percent of cyanobacterial reads (ANOVA, F = 12.79, *P* = 0.007) after a Bonferroni correction using a *P* ≤ 0.016 significance cutoff. *Post hoc* multiple comparison testing showed significantly more cyanobacterial reads were found on average (±SE) in orange morphs (3.31% ± 0.77) and seawater (3.6% ± 0.19) compared to brown color morphs (0.95% ± 0.67) from the Bahamas (Tukey’s HSD *P* < 0.05). For corals this is further supported by the flow cytometry results.

### Flow Cytometry

**Figure 2:**
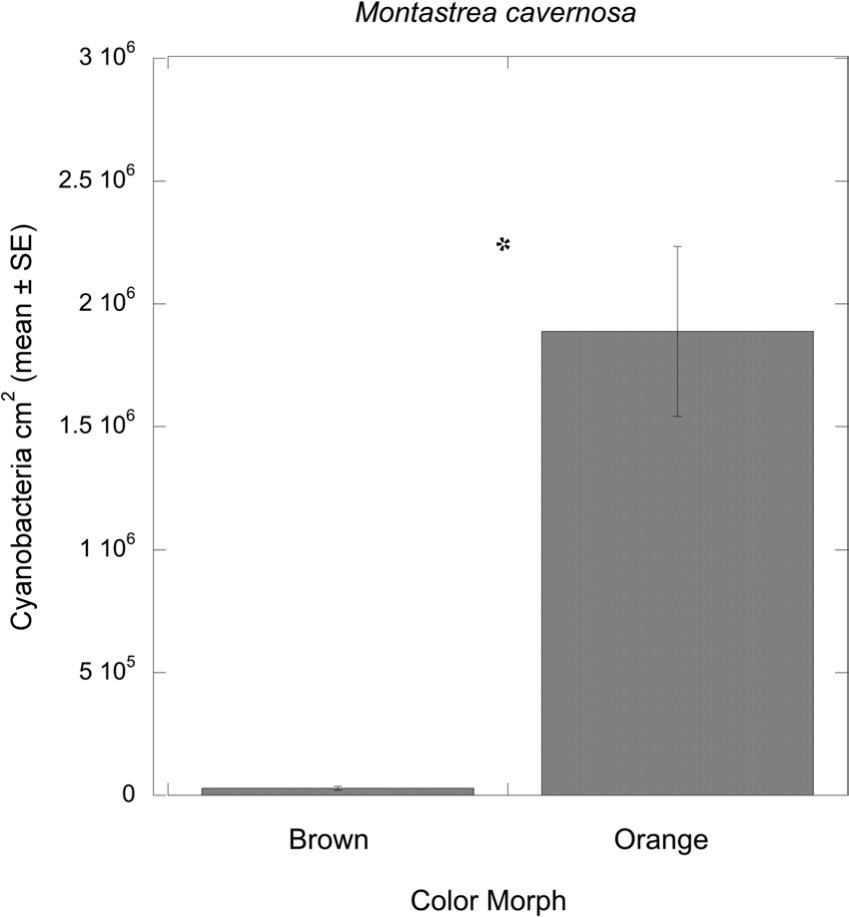
Results of flow cytometry analysis for cyanobacteria per unit area for color morphs of *Montastraea cavernosa*. *Indicates a statistically significant difference in phycoerythrin-positive cells (P < 0.0001).

The analysis of tissue homogenates using flow cytometry showed a distinct phycoerythrin signature and a size range of 1.0 to 3.0 µm diameter as previously reported^16^ in the orange morph of *Montastraea cavernosa* compared to the brown morph (Fig. S1). The number of cyanobacteria (i.e., phycoerythrin and chl *a*-positive cells) were significantly (ANOVA: F = 28.94, P < 0.0001) different between color morphs (Fig. 2) with orange morphs of *M*. *cavernosa*, normalized to surface area, averaging 1.9 × 10^6^ cells cm^−2^ while brown morphs averaged 2.9 × 10^4^ cells cm^−2^.

### Transcriptome Assembly

Illumina sequencing produced between 17,005,983 and 20,874,498 paired-end read pairs for the brown morph and between 20,823,031 and 22,747,092 paired-end read pairs for the orange morph of *Montastraea cavernosa*, for a total of 122.5 million reads from fragments averaging 240 nucleotides in length (European Nucleotide Archive ID PRJEB18062). After filtering out contaminants and putative assembly artifacts, the reads were assembled into 74,978 transcripts corresponding to *M*. *cavernosa* and 41,385 corresponding to *Symbiodinium* spp. (DRYAD doi:10.5061/dryad.v2g01), of which 38,967 were annotated by at least one method. A total of 11,911 transcripts were identified as putative members of the *M*. *cavernosa* microbiome (DRYAD doi:10.5061/dryad.v2g01). BUSCO and TransRate evaluations were conducted on the *M*. *cavernosa* transcriptome (Tables 1 and 2). These quality control measures show that the *M*. *cavernosa* transcriptome is structurally sound, and contains a high proportion of expected transcripts.

**Table 1:** Quality control statistics for the assembled transcriptome: BUSCO metrics include statistics regarding the number of universal vertebrate single copy orthologs found in the assembly.

	BUSCO		
	Complete	Duplicated	Fragmented
Montastrea_v1.0.2.fasta	72.3%	23.2%	8.9%

72.3% of the Metazoan BUSCOs were identified as full length (complete) sequences, while 8.9% were found to be fragmented. 23.2% were found in greater than one copy in the *Montastrea* transcriptome.

**Table 2:** Quality control statistics for the assembled transcriptome: TransRate metrics are derived from mapping RNAseq reads to the assembly, with higher scores indicating a higher quality assembly.

	TRANSRATE					
	Score	Number of Contigs	Assembly Size	Percent Mapping	Percent Bases Uncovered	Percent Contigs Lowcovered
Montastrea_v1.0.2.fasta	0.61	74,978	122.6 Mb	91	1	1

A score of 0.61 ranks this assembly higher than the majority of other published transcriptomes, with 91% of reads mapping, and only 1% of bases uncovered (no read support) and 1% contigs low-covered (mean per-base read coverage of <10).

### Montastraea cavernosa Transcriptomes

**Figure 3:**
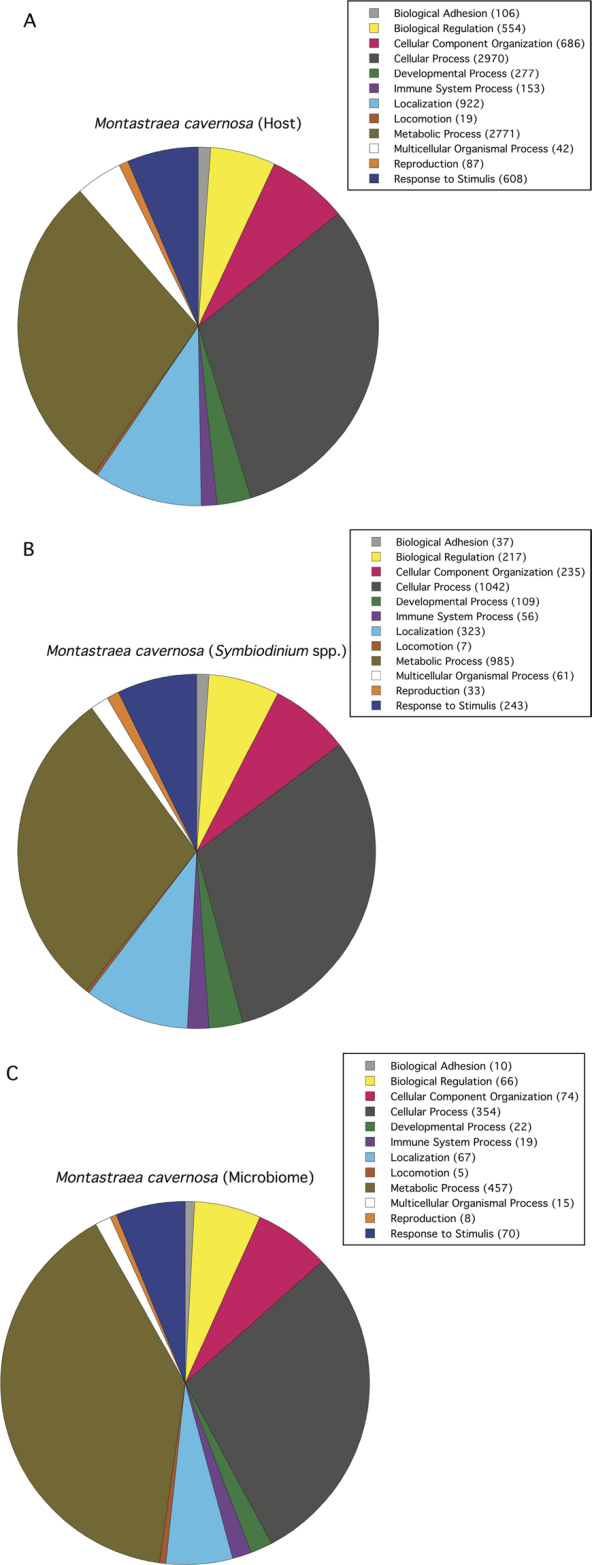
*Montastraea cavernosa* transcriptomes. Functional classification (biological process) for (**A**) host (total number of genes = 617, total number of process hits = 999), (**B**) *Symbiodinium* spp. (total number of genes = 178, total number of process hits = 328) and (**C**) Microbiome (total number of genes = 178, total number of process hits = 328). Number in parentheses next to GO groupings indicates number of genes. Pie charts represent the percent of gene hits against total process hits for each GO group.

Using the Gene Ontology (GO) in PANTHER (GO Biological Process) the putative transcripts for the *Montastraea cavernosa* host were assigned to 9,195 genes in twelve GO functional categories (Fig. 3A). Both Cellular Processes and Metabolic Processes made up the greatest proportion of identified genes encompassing over 50% of identified genes. The putative transcripts from the *Symbiodinium* spp. metatranscriptome were assigned to 3,348 genes with Cellular Processes and Metabolic Processes accounting for over 60% of identified genes (Fig. 3B). Lastly, the transcripts from the microbiome metatranscriptome of *M*. *cavernosa* were assigned to 1,167 genes with Cellular Processes and Metabolic Processes accounting for 70% of identified genes (Fig. 3C).

Looking specifically at transcripts related to Apoptosis, Pigment production and Immune functions we find that the *Montastraea cavernosa* host transcriptome contains multiple elements of the vertebrate apoptosis pathway including caspases, Bcl-2, DNA topoisomerase and MAP-kinase activating death domain as well as multiple transcripts of other stress related genes such as heat shock transcription factor and heat shock proteins (Table S1). In the Pigmentation category opsin gene transcripts were identified, as were transcripts associated with heme synthesis and melanosome maturation (Table S1). The Stress and Immune categories contained the greatest number of transcripts and included transcripts associated with DNA repair, enzymatic quenching of reactive oxygen species (ROS), apoptosis, cytokines, heat shock proteins and ubiquitination, intermediate metabolism, CO_2_ hydration/dehydration, cell cycle and regulation of transcription (Table S1).

Similar to the host transcriptome the *Symbiodinium* spp. metatranscriptome also identified transcripts such within the Apoptosis category such as caspases and DNA topoisomerase while the Pigmentation category is represented by transcripts from genes involved in heme synthesis (Table S1). Again, the Stress and Immune categories contained the greatest number of transcripts including representatives of DNA synthesis and repair, enzymatic quenching of ROS, apoptosis, heat shock proteins and ubiquitination, intermediate metabolism, CO_2_ hydration/dehydration, cell cycle, purine metabolism and regulation of transcription (Table S1).

Lastly, the microbiome metatranscriptome of *Montastraea cavernosa* has far fewer annotated transcripts than either the host or *Symbiodinium* spp. compartments (11,911 compared with 74,978 and 41,385, respectively) but those transcripts identified in the Apoptosis category include MAP-kinase activating death domain and apoptotic chromatin condensation inducer while the Pigmentation category is, like the *Symbiodinium* spp. compartment, represented by transcripts from genes involved in heme synthesis (Table S1). The Stress category contained the greatest number of transcripts and includes representatives of carbon metabolism, intermediate metabolism, DNA synthesis and repair, enzymatic quenching of ROS, apoptosis, heat shock proteins and ubiquitination, cell cycle, purine metabolism and regulation of transcription (Table S1). Immune functions were represented by enzymatic quenching of ROS, DNA synthesis and repair, apoptosis, heat shock proteins and ubiquitination and regulation of transcription (Table S1).

### Differential Expression Analyses

**Figure 4:**
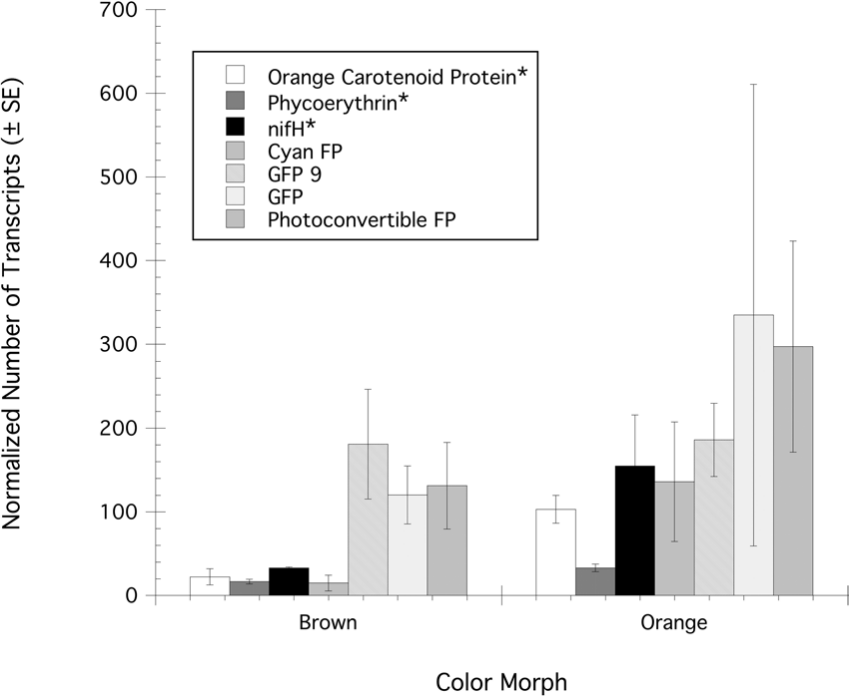
Differential expression of normalized transcripts for specific functional genes related to cyanobacterial pigments, nitrogen fixation and host fluorescent pigments. Where applicable * indicates a significant difference (P < 0.05) for each gene between the brown and orange morphs of *Montastraea cavernosa*.

The analysis of the differential expression of specific genes related to cyanobacterial pigments, nitrogen fixation and host fluorescent pigments showed that orange carotenoid protein (ANOVA: F = 17.66, P = 0.014), phycoerythrin (ANOVA: F = 8.24, P = 0.045) and *nifH* (ANOVA: F = 12.85, P = 0.023) from the metatranscriptome of the prokaryotic component of the microbiome were significantly up-regulated in orange versus brown morphs (Fig. 4) while no significant differences were observed for any fluorescent protein between color morphs in the host transcriptome (Fig. 4).

**Figure 5:**
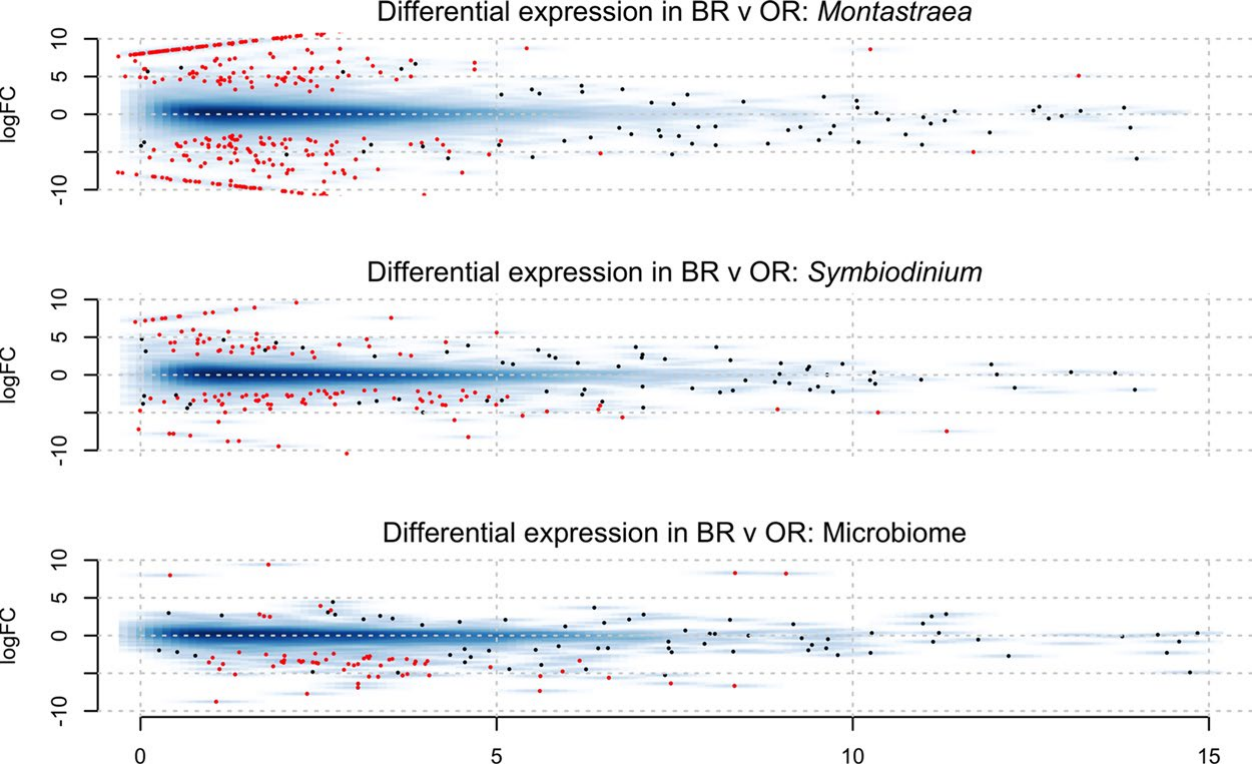
Global differential expression analysis on normalized count data using the edgeR pipeline; red dots are significantly different between brown and orange morphs of *Montastraea cavernosa* while blue dots are not significant for the host, *Symbiodinium* spp and microbiome.

Global patterns of differential expression between the brown and orange morphs of *Montastraea cavernosa* were analyzed using the software package edgeR. Specifically, we tested for differential expression in each transcript set from *M*. *cavernosa*: those putatively belonging to the coral host, *Symbiodinium* spp. and microbiome, which include bacteria and fungi. The analysis of the coral host fraction resulted in the discovery of 302 differentially expressed transcripts (Fig. 5); 136 more highly expressed in the orange morph and 166 more highly expressed in the brown morph (Table S3). Of these, 184 were annotated using the OrthoDB database, 191 were matched to proteins in the UniProt database, and 145 were annotated using the Pfam database (Table S2). Over 67% of the differentially expressed transcripts were annotated using at least one method. Genes exhibiting significantly greater expression in orange morphs included those from the immune response, response to oxidative stress and regulation of apoptosis (e.g., Bcl-2). Analysis of the *Symbiodinium* spp. compartment yielded 129 differentially expressed genes (Fig. 5), with 79 more highly expressed in the *Symbiodinium* spp. from the orange morph, and 50 more highly expressed in the *Symbiodinium* spp. from the brown morph (Table S3). Notably, these differentially expressed transcripts were more poorly annotated, with only 53 matching Uniprot accessions, 50 matching OrthoDB entries, and 27 matching a Pfam database entry: the only annotated genes more highly expressed in orange morphs were involved in apoptosis (e.g., Death Domain). Lastly the analysis of the prokaryotic microbiome yielded 79 differentially expressed genes (Fig. 5), with only 11 more highly expressed in the microbiome of the orange morph and the remaining highly expressed in the brown morph (Table S5). Of these only 23 were matched to known proteins in the Uniprot accessions, OrthoDB and Pfam databases (Table S5). Notable amongst these was the higher expression of psbD (D2 protein of photosystem II in cyanobacteria) from the orange morphs of *M*. *cavernosa* (Table S4).

### Symbiodinium spp. genotyping

**Figure 6:**
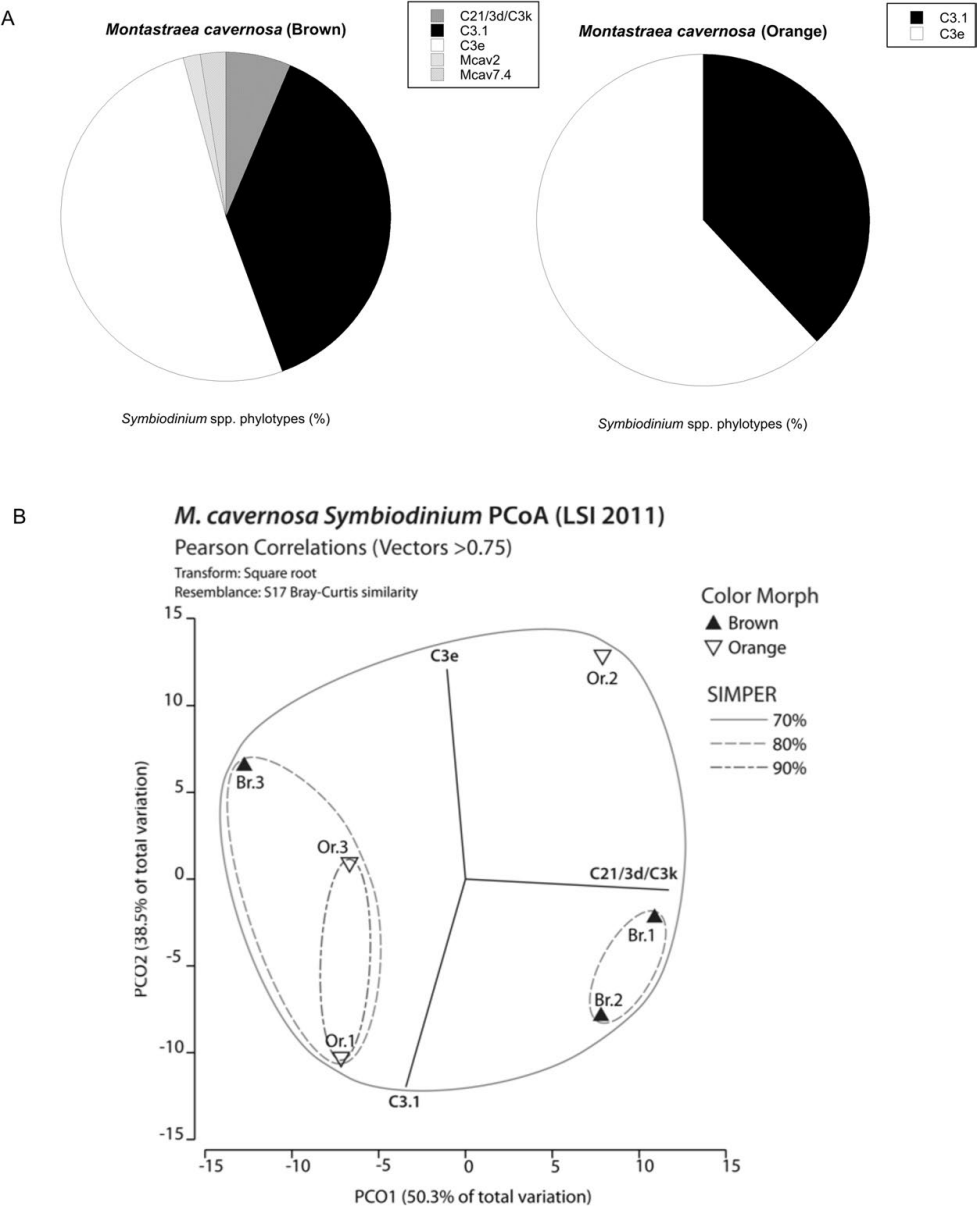
*Symbiodinium* spp. phylotyping . (**A**) Pie chart representing average percent contribution of the most dominant ITS phylotype relative to the total abundance in the color morphs of *Montasreaea cavernsa*. (**B**) Principal coordinate analysis (PCoA) plot based on square-root transformed Bray-Curtis similarities for *Symbiodinium* spp, ITS phyotypes of *Montastraea cavernosa*.

A total of 106 *Symbioindium* spp. ITS clones were sequenced and clustered (DRYAD doi:10.5061/dryad.v2g01) into five distinct phylotypes, all within clade C (Fig. 6A). The *Symbiodinium* spp. phylotypes C3.1 and C3e dominated the community composition of both color morphs (Fig. 6A). Many sequences were identified as being closely related to those previously found in *Montastraea cavernosa* (designated “Mcav”)^16^. Color morph was not a significant factor structuring *Symbiodinium* spp. communities (PERMANOVA, Pseudo-F = 0.331, *P* (perm) = 0.9), demonstrated by both overlap and dispersion in ordination plots using PCoA (Fig. 6B). The *Symbiodinium* spp. phylotypes C21/3d/C3k, Mcav2 and Mcav7.4 were only found in brown morphs (Fig. 6a).

### Population Genetics of Montastraea cavernosa Color Morphs

For the AFLP analysis 86 polymorphic loci were identified for analysis^27^. Brown and orange *Montastraea cavernosa* from LSI formed two genetically distinct populations at all depths including the depth of collection for this study. In the fitted CCA model, the constraints accounted for 15% of the AFLP variance. Subsequent ANOVA-like permutation tests indicated that the constraint terms, CCA model and axes explained more AFLP variance than expected by chance with color morph and depth significantly affecting the AFLP variance (Fig. S2A,B; Table S5). Also, our analysis using AFLPOP correctly assigned orange samples to the “orange” population 87.5% of the time, whereas brown samples were correctly categorized 50.5% of the time.

AMOVA was then used to compare the relative levels of genetic differentiation between brown and orange colonies at LSI, relative to those between brown colonies from the different locations surveyed in Brazeau *et al*.^27^. Brown samples from LSI, Little Cayman and San Salvador had an average Φ_ST_ of 0.091 (*P* = 0), with Φ_ST_ values for individual locations varying from 0.0809 to 0.1165 (Table S5). These average Φ_ST_ values indicate that there is less genetic differentiation between populations from different locations than was observed between brown and orange morphs at LSI from all depths including 15 m.

## Discussion

Here we have shown that the brown and orange morphs of *M*. *cavernosa* represent two significantly different genetic populations. The color morphotypes were dominated by the same phylotypes of their primary endosymbiont, *Symbiodinium* spp., with three distinct phylotypes found only in brown colonies, but these differences in *Symbiodinium* spp. communities are not statistically significant. These color morphs occur side-by-side so differences in environmental influences on host-microbe interactions would be predicted to be minimal^28^, suggesting that any observed differences are driven by the genetic differences between hosts and potentially *Symbiodinium* spp. composition. While we are beginning to understand that different coral species may harbor different “core” microbiome communities^14,15^ differences in host populations and *Symbiodinium* spp. communities may also influence the community structure of their prokaryotic microbiome^14^. In this study no significant differences in the overall composition of the microbiome community between color morphs of *M*. *cavernosa* was observed but there are differences in the percentage of reads assigned to cyanobacteria, with significantly more found in the orange morphs than in the brown morphs. This is also consistent with the flow cytometry results presented here, and in previous studies^18^.

If we target specific genes in the metatranscriptome of the microbiome we again see results consistent with previous work; the differential expression analyses of *nif*H and phycoerythrin as well as orange carotenoid protein shows significantly more transcripts for these genes in orange morphs than in brown morphs. Orange carotenoid protein is unique to cyanobacteria where it plays the dual role as a photoreceptor in the blue region of the spectrum, and in photoprotection of photosystem II (PSII) from high irradiances by triggering non-photochemical quenching (NPQ) in the phycobilisome^29^. The mechanism is believed to involve a decrease in the functional absorption cross-section of PSII that then reduces the amount of light energy being absorbed and transferred from the phycobilisome to the reaction centers^30^. Additionally, over 50% of the *nif*H transcripts in orange morphs were cyanobacterial in origin while 34% were of cyanobacterial origin in the brown morph. We also showed that the genes for multiple fluorescent proteins, including representatives of the cyan, brown and photoconvertable types, express a greater number of transcripts in orange versus brown morphs but these differences were highly variable, and not significant. In part, this could be a function of the low sample size analyzed here for this study and this aspect needs to be examined more fully. Previous work, utilizing confocal microscopy, also identified all three types of fluorescent proteins in the orange morphs of *M*. cavernosa^17^. Nonetheless, it is clear that orange morphs contain pigment genes for both host fluorescent and cyanobacterial pigments in greater abundance than brown morphs, and orange morphs contain over twice as many photoconvertible fluorescent protein transcripts as phycoerythrin and orange carotenoid protein transcripts combined, suggesting that fluorescent proteins may be responsible for more of the observed fluorescence than the *in hospite* cyanobacteria for these corals. The underlying basis for any changes in the expression of either host or cyanobacterial pigments in any single coral is presently unknown but one important factor is the underwater light field. Lesser *et al*.^19^ reported a significant increase in orange morphs, as a percent of the total population, with increasing depth. The presence of more orange morphs with increasing depth is consistent with both low light photoacclimatization of cyanobacteria that would increase phycoerythrin synthesis, and decreasing irradiances of long wavelength ultraviolet, and blue wavelengths, which are required to convert the photoconvertible fluorescent protein from green to red^31^. Additionally, the gene expression and protein concentrations of both green and red fluorescent proteins have been shown experimentally to decrease as irradiance decreases^32,33^. Either of these scenarios could lead to a decrease in the accumulation of photoconvertible fluorescent protein with increasing depth and would favor the presence of cyanobacteria and their associated photosynthetic pigments.

The corals described here were collected from 15 m where irradiances are high, and the two prevailing theories for the expression of fluorescent proteins in shallow water has been photoprotection or as an accessory pigment for photosynthetic light capture^34^. But biophysical and molecular evidence has shown that photoprotection or the enhancement of photosynthesis by fluorescent proteins, if it exists, is negligible^35,36^. One function of fluorescent proteins that is indirectly related to irradiance is the quenching of ROS^37,38^, but its importance in this role relative to more effective and efficient antioxidant systems (i.e., enzyme based) in corals and their multiple symbiotic partners is unknown even though the production of ROS, like the expression of fluorescent proteins, increases with increasing irradiance^39^.

The transcriptome/metatranscriptomes of *Montastraea cavernosa* described here are comparable to similar studies on *M*. *cavernosa* ^40,41^. But comparing the transcriptomes of brown and orange morph host tissues revealed many phylogenetically conserved transcripts related to the immune response, response to oxidative stress and regulation of apoptosis, not acquired by horizontal gene transfer^41^, that were upregulated in orange morphs compared to brown morphs. Since both morphotypes were collected at midday under similar conditions of irradiance, nutrients and temperature these differences are probably not the result of these, or other, environmental drivers. Additionally, transcriptional differences driven by circadian rhythms should not strongly drive differences in these corals based on the time and place of collection^41^. One hypothesis for this pattern of differential expression in the host tissues is that the presence of additional, different, or more numerous symbionts in the microbiome of orange morphs presents an immunological challenge requiring more active surveillance and regulation^42^. Notably, the *Symbiodinium* spp. metatranscriptome in orange morphs showed an increased expression of transcripts involved in apoptosis. These *Symbiodinium* are also exposed to a supply of fixed nitrogen and have been shown to grow at a faster rate in orange morphs compared to brown morph, yet both hosts maintain the same constant biomass of symbionts^19^. As a result, a parallel increase in the rate of apoptosis in *Symbiodinium* could maintain the observed constant density of symbionts despite a faster growth rate. These unique attributes of the symbioses could be a source of positive selection and the population differentiation between brown and orange morphs reported here.

Unlike the host there were far fewer transcripts differentially expressed between the *Symbiodinium* spp. communities of brown and orange morphs of *Montastraea cavernosa* and many were poorly annotated. Part of the reason may be the small differences between the *Symbiodinium* spp. communities and, as in the host, the similar environmental conditions under which the collection was made. *Symbiodinium* also has a relatively small genome compared to other dinoflagellates^43^, and while more genomic resources have been made available^44^ there are several features that can complicate the analysis of dinoflagellate genomes including their high GC content, non-conventional bases, and horizontally transferred genes. Finally, almost all dinoflagellate mRNAs are capped with a highly conserved 22-bp spliced-leader sequence that appears to be important in regulating translation^45^. This sequence has recently been utilized as a target for PCR primers to amplify cDNAs^46^. The minimal changes in transcription observed in many studies of dinoflagellates suggest that either much of the gene regulation occurs post-transcriptionally^47^, or that specific targeting of the spliced-leader sequences may be necessary to increase the recovery of many transcripts^46^.

The differential expression of prokaryotic genes in the metatranscriptome provided no additional conclusive physiological differences between the brown and orange morphs of *Montastraea cavernosa* except in the form of another cyanobacterial marker, psbD (D2 protein of photosystem II in cyanobacteria), which adds support to the reported differential expression of targeted genes described above and other data presented here such as the number of cyanobacterial reads and flow cytometry data.

The analysis above provides an important examination of the genetic, metagenetic and transcriptomic underpinnings for the differences in the co-occurring color morphs of the coral *Montastraea cavernosa*. We present a new set of methods utilizing easily accessible tools for examining the coral holobiont from a holistic perspective. Future studies would most certainly benefit from greater spatial replication (e.g. site and depth) and sample size as recommended by Todd *et al*.^48^. Additional supportive studies such as NanoSIMS, RT-qPCR of specific genes and apoptosis assays (e.g., TUNEL assay) should be included as well. However, the annotated transcriptome/metatranscriptome assemblies presented here will be of broad use for the study of the biology of corals and specifically for continuing studies on the causes for color variability in corals, and their functions.

## Methods and Materials

### Sample Collection and DNA Extraction

Samples from orange (n = 3) and brown (n = 3) colonies of the coral *Montastraea cavernosa* were collected with a hammer and chisel on SCUBA from North Perry Reef, Lee Stocking Island (LSI), Bahamas (23°47′0.03′′N, 76°6′5.14′′W) at a depth of 15 m midday in August of 2011. Additionally, three replicate water samples (4 l) were collected from 1 m above the reef substrate. Coral and water samples were transported to the laboratory in covered coolers filled with seawater. Corals were then gently airbrushed while being held upside down with 0.2 μm filtered seawater (FSW) from a distance of approximately 15 cm to remove mucus and loosely associated bacteria^19^. Water samples were vacuum filtered onto 0.2 µm filters (Millipore) and stored at −20 °C. Subsamples of all corals were then placed in saline DMSO buffer^49^ or RNALater buffer (Ambion), frozen at −20 °C, and transported to the University of New Hampshire. Genomic DNA was extracted from each coral and filtered seawater using a PowerSoil DNA extraction kit (MoBio) while a hexadecyltrimethylammonium bromide (CTAB) protocol was used to extract additional DNA from filters. DNA from CTAB extractions and PowerSoil extractions of filters were pooled in equal amounts.

### 16S rRNA Pyrosequencing

The V5-V6 hypervariable regions of the 16S rRNA gene were amplified with PCR using universal primers U789F and U1068R fused with GS FLX Titanium adapters A or B respectively, and a 10 bp barcode unique to each sample as described by Lee *et al*.^50^ and cover 94.8 to 97.7% of publicly available sequences, including cyanobacteria. Triplicate PCR reactions were performed on each coral and seawater sample as previously described^51^ with the pooled products for each sample separated by gel electrophoresis and purified with a QiaQuick gel extraction kit (Qiagen, Valencia, CA) followed by AMPure XP beads (Beckman Coulter, Danvers, MA). DNA concentrations were measured with a Qubit dsDNA high sensitivity assay (Invitrogen, Carlsbad, CA) and amplicons were pooled in an equimolar fashion. Emulsion PCR and bidirectional multiplex 454 pyrosequencing was carried out using Titanium FLX chemistry at the W.M. Keck Center for Comparative and Functional Genomics at the University of Illinois at Urbana-Champaign.

Sequences were processed through the Quantitative Insights into Microbial Ecology (QIIME) pipeline v 1.9.1^52^. Briefly, sequences of length <200 bp, with ambiguous base calls or homopolymer runs exceeding 8 bp were removed. Sequences were aligned against the August 2013 curated Greengenes reference database (gg_13_8_otus; http://browngenes.secondgenome.com/alignment). Operational taxonomic units (OTUs) were defined using PyNAST after removal of singleton sequences and clustering at 3% divergence (97% similarity). Chimeric artifacts were also removed using UCHIME^53^. OTUs were taxonomically classified using a BLAST-based method against the August 2013-curated Browngenes database, and compiled at each taxonomic level into a counts file. Any sequences that were classified as Mitochondria, Eukaryotic or Chloroplast as well as any sequences of unknown origin were filtered out of the dataset. Sequence counts were normalized to a proportion to account for variation in sampling depth by dividing by the total library size (sampling depth of 2200 sequences per sample).

OTU tables were built from QIIME-generated.biom files and Bray-Curtis distance matrices were built to examine patterns of community structure and visualized using principal coordinate analyses (PCoA) and differences between color morphs were then tested for statistical significance with permutational analysis of variance (PERMANOVA, using 9,999 permutations) determined whether spatial separation between color morphs was statistically significant. The above multidimensional statistical analyses were performed in PRIMER V7 with the PERMANOVA+ add-on (PRIMER-E Ltd., Devon, UK).

### Flow Cytometry

Samples of ~1 cm^2^ from both orange (n = 10) and brown (n = 10) morphs of the same population of *Montastraea cavernosa* described above were collected and each sample lightly airbrushed as described above to remove excess mucous and associated microbiota. The orange (n = 3) and brown (n = 3) colonies described above for the analysis of 16S rRNA were included in this larger sampling of corals. Corals were then airbrushed (80 psi, <1 cm distance to coral) to remove coral soft tissues using a small volume (1–2 ml) of filtered seawater. The samples were homogenized with a handheld homogenizer to shear mucous and homogenates were then fixed at a final concentration of 0.5% with paraformaldehyde and frozen at −50 °C. Samples were sent frozen to the Bigelow Laboratory for Ocean Sciences Flow Cytometry Facility. Each sample was diluted 1:100 with FSW, stained with PicoGreen, and analyzed using a Becton Dickinson FACScan flow cytometer equipped with a 15 mW, 488 nm, air-cooled Argon ion laser. Simultaneous measurements of forward light scatter (FSC, relative size), side scatter (SSC), PicoGreen fluorescence (515–525 nm), chlorophyll fluorescence (>650 nm), and phycoerythrin fluorescence (560–590 nm) were made on all samples as previously described^18^. PicoGreen fluorescence was used as an initial screen to distinguish cells from non-cells, and then fluorescent beads and cultured cyanobacteria were used to identify and calibrate the flow cytometery to gate on cyanobacteria exhibiting both chlorophyll and phycoerythrin fluorescence. The surface area normalized concentrations of cyanobacteria were log transformed and analyzed using a one-way ANOVA with color as a fixed factor.

### Analysis of the Transcriptome/Metatranscriptome of Montastraea cavernosa

Total RNA was extracted from the same samples of each color morph (n = 3 brown, n = 3 orange) as described above for the analysis of 16S rRNA. Samples were preserved in RNAlater (Ambion) and an RNAqueous extraction kit (Invitrogen) was used to extract RNA according to the manufacturer’s instructions except for the following modifications: tissue was homogenized by bead beating in 750 μl of lysis buffer with 0.3 g of 160 μm glass beads for 3 min at maximum speed in a Vortex Genie 2 with Turbomix attachment (Scientific Industries, Inc.), and total RNA was eluted twice with 50 μl and 15 μl of elution solution. DNA was removed using the “rigorous” protocol for the TURBO DNA-free kit (Invitrogen), and complete digestion of DNA was confirmed by the absence of visible bands from PCRs amplifying cnidarian actin and Eubacterial 16S rRNA genes. Samples were sent to the W. M. Keck Center for Comparative and Functional Genomics at the University of Illinois for the preparation of libraries and Illumina sequencing. Eukaryotic rRNA was removed from 1 μg of total RNA from each sample using subtractive hybridization with a RiboMinus Eukaryote kit (Invitrogen) according to the manufacturer’s instructions. Libraries were prepared with TruSeq RNAseq sample prep kits (Illumina), pooled in equimolar concentrations, and quantitated with qPCR. The six samples were multiplexed and sequenced on a HiSeq. 2000 using a single lane of 100 bp paired-end sequencing.

### Sequence Quality Control and Assembly

The raw sequence reads corresponding to the two color morphs were error corrected using the software Lighter version 1.04 (https://goo.gl/XQYQEq) Lighter was selected as the error correction package based on its low frequency of introduction of erroneous changes to the nucleotide sequence^54^. The error-corrected sequence reads were adapter and quality trimmed (threshold of Phred <2) with Trimmomatic version 0.32 (https://goo.gl/vlHjeV) following recommendations from^55^. Reads from each color morph were assembled together to create a joint assembly of coral transcripts using Trinity version 2.20 and BinPacker version 1.0.0, on a Linux workstation with 64 cores and 1Tb RAM (https://goo.gl/Q8pWUu). We used flags to indicate the stranded nature of sequencing reads and set the maximum allowable physical distance between read pairs to 999 nt. These two assemblies were merged together (https://goo.gl/64xHJF) to create a final assembly using the software package transfuse (https://github.com/cboursnell/transfuse).

Because this final assembly contains transcripts corresponding to the coral host and it’s microbial community it includes both the prokaryotic microbiome and the intracellular symbiont *Symbiodinium* spp., each of which may be contributing to different aspects of the coral phenotype. As a result we separated transcripts originating from these compartments that make up the coral holobiont^13^. To accomplish this, we used a BLASTX procedure, after constructing a large merged protein database, consisting of all RefSeq protein datasets corresponding to specific taxonomic groups defined as Plants, Bacteria, Archaea, Fungi, Protozoa and Invertebrates available from the NCBI, as well as a *Symbiodinium* sp. proteome available at http://marinegenomics.oist.jp/symb/download/symbB.v1.2.augustus.prot.fa.gz. We searched the primary assembly using an e-value of 1 × 10^−5^. Transcripts matching cnidarians were retained as putative *Montastraea cavernosa* transcripts, transcripts matching *Symbiodinium* spp. were filtered into a separate assembly, while transcripts matching other taxa, including bacteria and fungi were retained in a third assembly file. While this approach may inappropriately classify some sequences, particularly in cases where reference sequences are misclassified or are the result of horizontal gene transfer; this approach has a high probability of accurately classifying the vast majority of transcripts.

The quality of the filtered transfuse-merged *Montastraea cavernosa* assembly was evaluated using TransRate version 1.01. TransRate generates quality statistics based on a process involving mapping sequence reads back to the assembled transcripts. Transcripts supported by properly mapped reads of a sufficient depth were judged to be of high quality. Then we evaluated transcriptome completeness via use of the software package BUSCO version 2. BUSCO searches against a database of highly conserved single-copy Metazoan genes where high quality complete transcriptomes are hypothesized to contain the vast majority of these conserved genes.

### Assembled Sequence Annotation and Differential Expression

The filtered *Montastraea cavernosa* and *Symbiodinium* spp. assemblies were annotated using the software package dammit version 0.2.9 (https://github.com/camillescott/dammit). Dammit coordinates the annotation process, which involves the use of BLAST, TransDecoder (http://transdecoder.github.io), and HMMER (http://hmmer.org), to search the Pfam, OrthoDB and Uniref90 databases. Additionally, we searched a database of transporter proteins, available at www.tcdb.org. To examine the differential expression of specific genes related to nitrogen fixation (*nif*H), host fluorescent proteins and pigment genes of cyanobacteria we used BLAST of known gene sequences from representative taxa (Table S6) to identify contigs in the transcriptome of the host or metatranscriptome of the microbiome from all samples of both color morphs. The normalized counts of identified transcripts for each functional gene was log transformed and analyzed using a one-way ANOVA with color as a fixed factor. To identify patterns of global gene expression we quasi-mapped the reads from each individual to the reference assemblies (*M*. *cavernosa*, *Symbiodinium* spp., and the microbiome) using Salmon version 0.7.2 (https://combine-lab.github.io/salmon). Analysis of differential expression was conducted on normalized count data based on trimmed mean of M-values (TMM) using the standard edgeR pipeline (http://bioconductor.org/packages/release/bioc/html/edgeR.html), while setting the false discovery rate (FDR) to 0.01 (1%).

### Symbiodinium spp. Phylotyping

Using the same DNA from the samples described above the internal transcribed spacer two region (ITS2) and flanking 5.8 S and 28 S regions of the rDNA of *Symbiodinium* spp. were sequenced and analyzed as previously described^56^. For each sample amplified DNA was purified, ligated into the pGEM-T Easy Vector, and transformed into *Escherichia coli* JM109 competent cells using a Promega cloning kit. Plasmids were purified and inserts sequenced from the T7 promoter primer at Functional Biosciences (Madison, Wisconsin). Additional plasmids were purified using PureYield Plasmid Minipreps (Promega, Madison, Wisconsin), and sequenced using a T7 promoter primer at the University of New Hampshire Hubbard Center for Genome Studies Sequencing Core Facility. Vectors were automatically trimmed from sequences, low-quality regions were removed, and ambiguous nucleotides were manually called where possible in Geneious 5.3.4. Cleaned sequences were then compared against the NCBI nr database using BLAST and sequences where the top hit was not the ITS2 region of *Symbiodinium* were removed. A clustering approach^57^ rather than traditional alignment to a reference database was used. Clustering is a replicable and well-regarded method in microbial ecology and has been previously used for identifying *Symbiodinium* spp. phylotypes using ITS2^58^. This approach is useful for describing *Symbiodinium* spp. communities because it can collapse intragenomic variants of ITS2 into a single OTU^59^. Scripts from the QIIME pipeline (version 1.7.0) were used to cluster sequences de novo into OTUs at a similarity threshold of 97%^57^, and align OTU sequences to a reference database of *Symbionidium* ITS2 sequences. OTU sequences that did not align and those flagged as chimeras by analysis with UCHIME were discarded. A phylotype was assigned to each OTU by BLAST comparison to the reference database, and in cases where multiple OTUs were classified as the same phylotype, a decimal and integer was added to each designation. Bray-Curtis similarity of square-root transformed read counts was analyzed using PERMANOVA and PCoA in PRIMER 7 using 9,999 permutations.

### Population Genetics of Montastraea cavernosa Color Morphs

Coral samples were collected at LSI to assess the population genetics of brown and orange morphs of *Montastraea cavernosa* across shallow to mesophotic depths^27^. Brown colonies from LSI were previously sampled (N = 3 at each depth) from 3, 10 15, 25, 30, 45, 60 and 75 m^27^. Orange colonies from LSI were also collected concurrently from 10, 15, 25 m (n = 6 at each depth) as well as 30 m (n = 5), 45 m (n = 4) and 60 m (n = 1) but are analyzed here for the first time. DNA extractions, amplified fragment length polymorphisms (AFLP), and analysis with analysis of molecular variance (AMOVA), population assignment program for AFLP data (AFLPOP) were done as described in Brazeau *et al*.^27^. Additionally, a constrained correspondence analysis (CCA) followed by multivariate ANOVA-like permutation tests were performed on the matrix of AFLP markers to test for relationships of depth and color with genetic states across AFLP markers using the R package *vegan* ^60^. Non-collinearity between constraints (color morph and depth) was evaluated using Variable Inflation Factors (*vegan*). We performed an AMOVA analysis and compared the results to previously published AFLP data on *M*. *cavernosa* from LSI, Little Cayman and San Salvador in the Bahamas^27^. We used fixation indices (Φ_ST_) to quantify the levels of genetic differentiation between brown and orange morphs of *M*. *cavernosa* at LSI, compared to the differentiation reported in previously published data on colonies of *M*. *cavernosa* from different geographic locations and depths. Data from Brazeau *et al*.^27^, however, could not be used in the same AMOVA analysis because the samples were not processed for AFLP in the same runs; therefore the resulting allele frequencies cannot be directly compared. However, Φ_ST_ values from AMOVA can be compared between the groups, with higher values indicating greater differentiation between groups or populations. P values for Φ_ST_ were estimated using a bootstrap and 999 iterations.

## Electronic supplementary material

Supplementary Information

Table S1

Table S2

Table S3

Table S4
