# Supplementary material for: Comparative Genomics of Color Morphs In the Coral Montastraea cavernosa
Source: Sci Rep. 2017 Nov 22;7:16039. doi: 10.1038/s41598-017-16371-9 (PMC5700045; doi:10.1038/s41598-017-16371-9)
Supplement: Supplementary file 1 — Supplementary Information [file 41598_2017_16371_MOESM1_ESM.pdf]

Supplementary Information

Comparative Genomics of Color Morphs in the Coral *Montastraea cavernosa*

Jessica K. Jarett[1†], Matthew D. MacManes[1], Kathleen M. Morrow[1], M. Sabrina Pankey[1], Michael P. Lesser[2]*

[1] Molecular, Cellular and Biomedical Sciences, University of New Hampshire, Durham, NH 03824, USA

[†] Current address: US Department of Energy, Joint Genome Institute, 2800 Mitchell Drive, Walnut Creek, CA 94598, USA

[2] School of Marine Science and Ocean Engineering, University of New Hampshire Durham, NH 03824, USA

*Corresponding author; mpl@unh.edu, 603-862-3442

Supplementary Table 1. List of genes used for BLAST searches for the coral *Monastraea cavernosa* transcriptome/metatranscriptome.

**Gene Name**	**Species**	**Genbank Acc. No.**
Water-soluble carotenoid pigment (OCP)	*Microcystis aeruginosa*	NC_010296.1
Phycoerythrin beta-subunit (*cpeB*)	*Synechocystis* sp.	AF169367.1
Phycoerythrin alpha-subunit (*cpeA*)	*Synechocystis* sp.	AF169367.1
Phycoerythrin lyase (*cpeY*)	*Synechocystis* sp.	AF169367.1
nifH	*Vibrio diazotrophicus*	U23650.1
nifH	*Crocosphaera watsonii*	AY221821.1
nifH	*Bradyrhizobium japonicum*	NC_004463.1
Cyan fluorescent protein	*Montastraea cavernosa*	AY056460.1
Green fluorescent protein isolate 9	*Montastraea cavernosa*	EU035535.1
Green fluorescent protein	*Montastraea cavernosa*	AF384683.2
Photoconvertible fluorescent protein	*Montastraea cavernosa*	EU035530.1

Figure S1. Flow cytometry bivariate graphs of phycoerythrin fluorescence versus sidescatter for A) orange morphs and B) brown morphs of *Montastraea cavernosa* showing the gating of the instrument (area enclosed in red lines) based on fluorescent beads of known size and fluorescent characteristics as well the characteristics of cultured cyanobacteria.

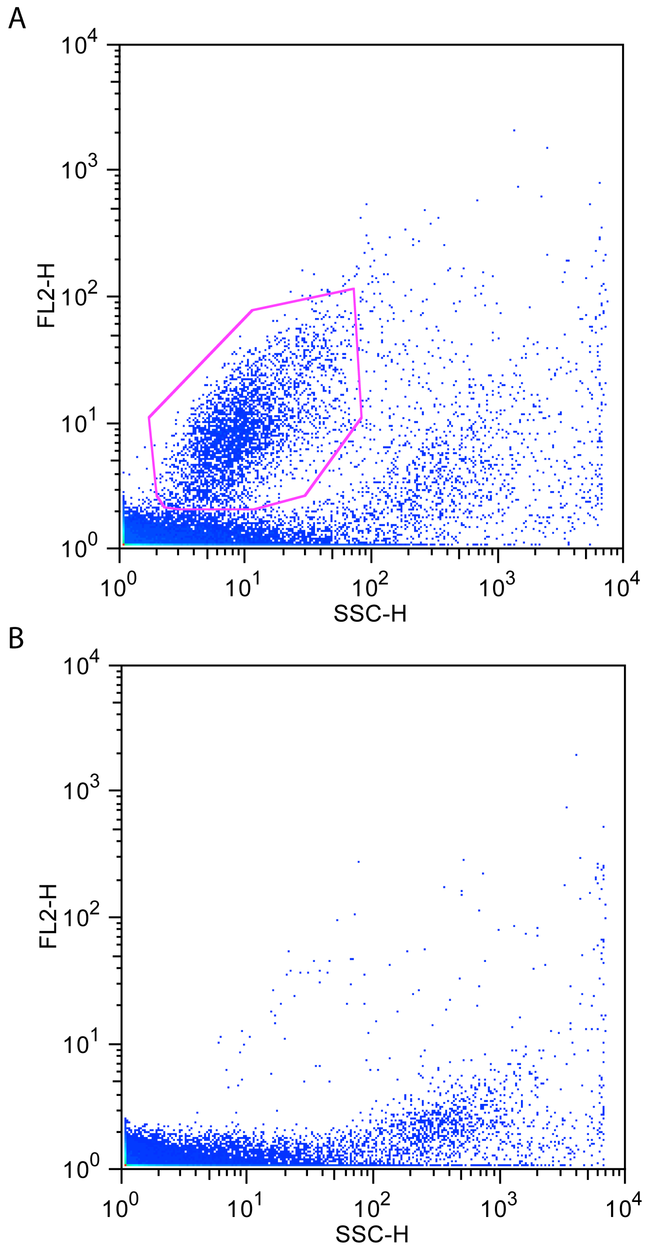

Figure S2 A. Ordination of AFLP markers (blue) relative to centroid positions of constraints (color morph and depth category) using their scores along first two CCA axes. MorphOR= orange morph; MorphBR = brown morph. DepthS = 3-10 m; DepthM = 15-25 m; DepthD = 30-45 m; DepthVD = 60-75 m.

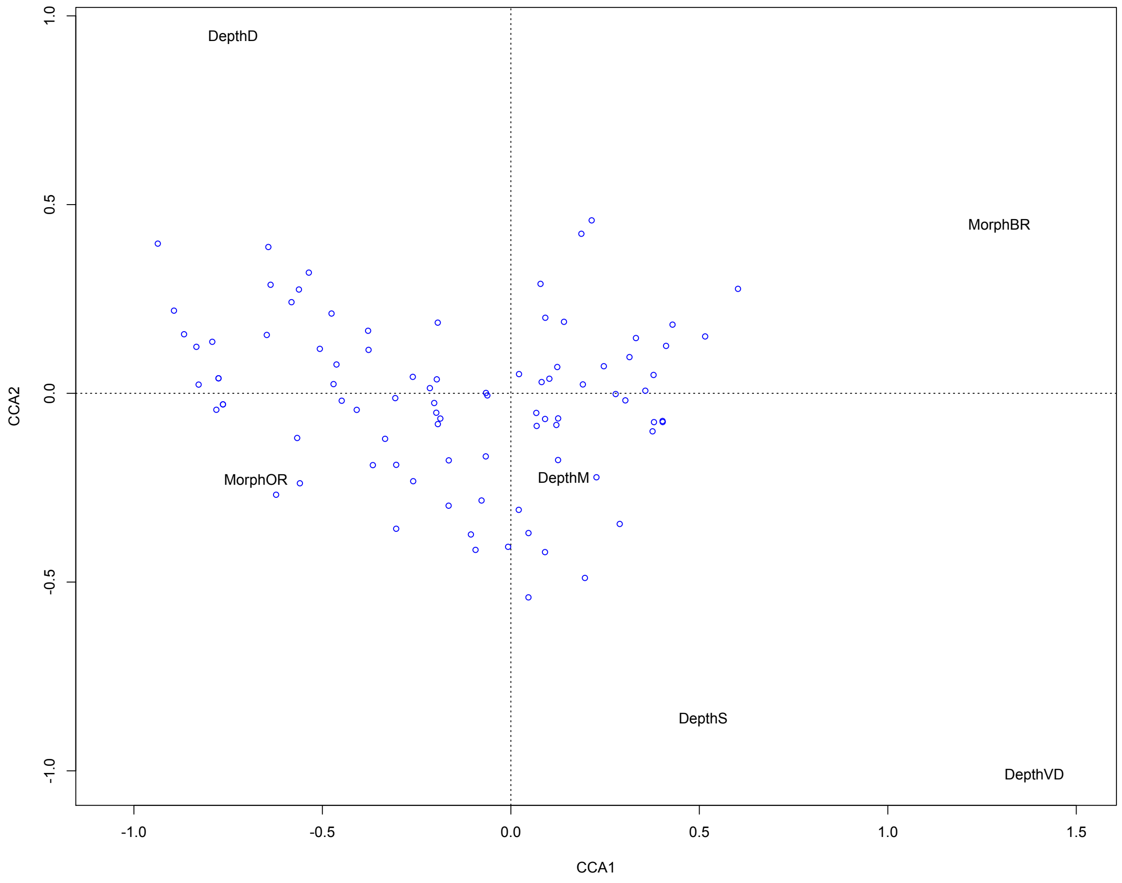

Figure S2 B. Ordination of *Montastraea cavernosa* samples using their scores along the top two axes extracted by Constrained Correspondence Analysis. Samples are colored by respective color morph state. Symbols reflect depth of collection in meters of seawater (m).

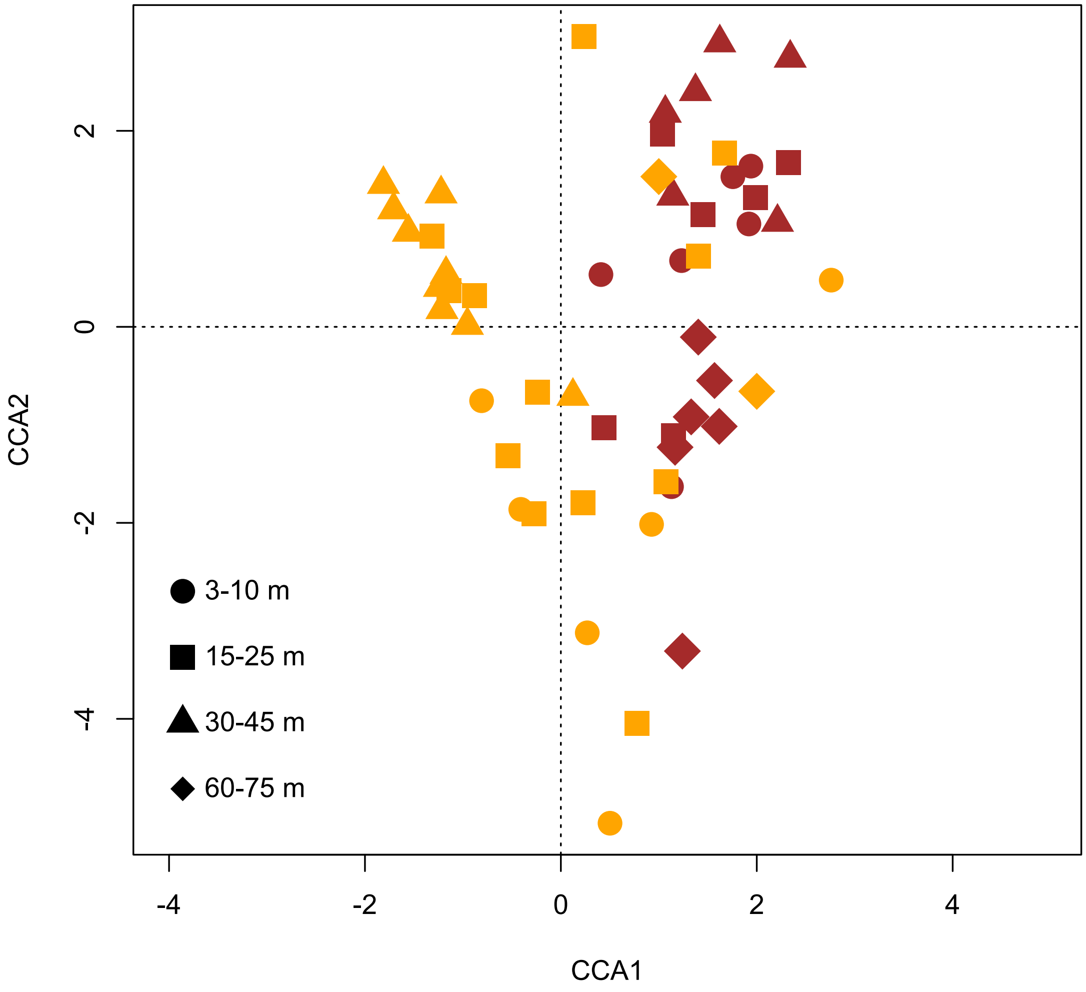

Table S5. Permutation tests on CCA model for *Montastraea cavernosa* AFLP data.

A. CCA model significance (999 permutations)

	df	ChiSquare	F	Pr(>F)
Model	4	0.22566	2.1179	0.001
Residual	47	1.2519		

B. CCA axis significance (999 permutations)

	df	ChiSquare	F	Pr(>F)
CCA1	1	0.13432	5.0427	0.001
CCA2	1	0.03633	1.364	0.057
CCA3	1	0.03161	1.1867	0.157
CCA4	1	0.0234	0.8785	0.59
Residual	47	1.2519		

C. CCA constraint terms (999 permutations)

	df	ChiSquare	F	Pr(>F)
Depth	3	0.13308	1.6654	0.005
Color Morph	1	0.09257	3.4755	0.001
Residual	47	1.2519		
